# The Use of a Mini-Bioreactor Fermentation System as a Reproducible, High-Throughput *ex vivo* Batch Model of the Distal Colon

**DOI:** 10.3389/fmicb.2018.01844

**Published:** 2018-08-10

**Authors:** Michelle M. O’Donnell, Mary C. Rea, Fergus Shanahan, R. P. Ross

**Affiliations:** ^1^APC Microbiome Institute, University College Cork, National University of Ireland, Cork, Ireland; ^2^Teagasc Food Research Centre, Moorepark, Fermoy, Cork, Ireland; ^3^Department of Medicine, University College Cork, National University of Ireland, Cork, Ireland; ^4^School of Microbiology, University College Cork, National University of Ireland, Cork, Ireland

**Keywords:** fecal fermentation, micro-Matrix, microbiota, mini-fermentation system, batch colon model

## Abstract

*Ex vivo* colon fermentation systems are highly versatile as models for analyzing gastrointestinal tract microbiota composition and functionality. *Ex vivo* colon models range in size and functionality from bench-top micro fermenters to large units housed in individualized cabinets. The length of set-up time (including stabilization periods) for each fermentation system can range from hours to weeks to months. The aim of this study was to investigate a single-use cassette mini-fermentation system as a reproducible batch model of the colon. The online data log from the cassettes (triplicate wells across four different cassettes, *n* = 12) was sensitive enough to identify real-time changes in pH, temperature, dissolved oxygen or liquid addition (sodium hydroxide) during the runs which could be addressed if an alarm set-point was triggered. The alpha diversity indices also showed little variation between cassettes with the samples clustering around the mean. The weighted beta diversity PCoA analysis illustrated that 95% of the variance between the samples was accounted for by the time-point and not the fermentation run/cassette used. The variation in taxonomic diversity between cassettes was limited to less than 20 out of 115 genera. This study provides evidence that micro-bioreactors provide some very attractive advantages as batch models for the human colon. We show for the first time the use of the micro-Matrix a 24-well sophisticated parallel controlled cassette-based bioreactors as a batch colon model. We demonstrated a high level of reproducibility across fermentation cassettes when used in conjunction with a standardized fecal microbiota. The machine can operate 24 individual fermentations simultaneously and are relatively cost effective. Based on next generation sequencing analysis, the micro-bioreactors offer a high degree of reproducibility together with high-throughput capacity. This makes it a potential system for large screening projects that can then be scaled up to large fermenters or human/animal *in vivo* experiments.

## Introduction

The use of batch and continuous fermentation systems has garnered a lot of attention in recent years as models to simulate the microbiota of the human gastrointestinal tract and in particular the human colon (Molly et al., 1993; Fooks and Gibson, 2003; Feria-Gervasio et al., 2011; Vamanu et al., 2013; Marzorati et al., 2014; Tanner et al., 2014; Fehlbaum et al., 2015). There are, however, fewmodels that can be truly used for high-throughput screening. Conventional *ex vivo* colon models are instrumented bioreactors that monitor and control temperature, pH and dissolved oxygen (DO) resulting in physiological colon conditions (Payne et al., 2012). The colon models are labor intensive to set-up and clean and often limited in the number of bioreactors available. The set-up and stabilization period of colon models can range from a couple of hours to a number of weeks (Feria-Gervasio et al., 2011; Rea et al., 2011). Williams et al. (2015) summarized the stabilization periods required or used with the different models (Williams et al., 2015) with stabilization periods of less than 48 h commonly used for batch fermentation systems. The time-consuming nature and number of vessels required can limit the number of experiments performed at any one time. However, as with microbial cell cultivation and bio-processing, micro-bioreactors can be used to facilitate *ex vivo* colon modeling and the rapid screening of a variety of parameters.

The agreed upon definition for a mini-bioreactor is any system whereby the working volume is 1–10 mL. Lattermann and Büchs (2015) co-authored a review summarizing some of the technologies behind the various mini and micro-bioreactors. Benefits of mini-bioreactors especially the micro-Matrix include integrated online monitoring and parallel control of fermentations using integrated sensors. Each well of the micro-Matrix cassette operates as a stand-alone bioreactor allowing for cassette-wide gradients of pH, dissolved oxygen and temperature. The pre-calibrated integrated pH and DO sensors remove the need to calibrate each individual well prior to beginning the fermentation which even with a smaller number of bioreactors is time difficult and time consuming.

The goal of our work was to demonstrate the use of a mini-fermentation system as a reproducible *ex vivo* batch colon model. The benefit of the micro-Matrix cassette (used in this study) is that up to 24 different conditions can be tested at any one time since each cassette houses 24 wells which can be inoculated with fecal slurry and maintained under physiological conditions. The results demonstrate that the mini-fermentation system provides real-time monitoring of pH, %DO and liquid addition enabling the user to carefully monitor any changes between control samples and test conditions. This is of great benefit to users who have a finite amount of fecal matter (in the case of infants for example) or allows a range of different interventions to be tested in parallel efficiently and economically.

## Materials and Methods

### Donor Recruitment

Donor recruitment and enrollment were approved by the Clinical Research Ethics Committee of the Cork Teaching Hospitals (protocol no. APC055). All donors completed a questionnaire demonstrating their willingness to participate in the study. All donors were healthy adults (23–50 year) who hadn’t taken an antibiotic for 6 months prior to donating a fecal sample.

### Frozen Standardized Inoculum and Fermentation Medium

The frozen, standardized inoculum (FSI) was prepared under strict anaerobic conditions as outlined previously (O’Donnell et al., 2016). A total of six donor fecal samples were used to prepare the FSI under anaerobic conditions with the resulting filtered slurry prep resuspended in 50 mM potassium phosphate buffer (pH 6.8) containing 25% glycerol and frozen at -80°C. The fermentation medium used was as outlined by Fooks and Gibson (2003), however, fructooligosaccharide (FOS, Raftilose Synergy 1, Beneo Orafti) replaced glucose as a carbon source (Fooks and Gibson, 2003). The FOS was added to the medium at 5% (w/v).

### micro-Matrix^TM^ Cassette Set-Up

**FIGURE 1:**
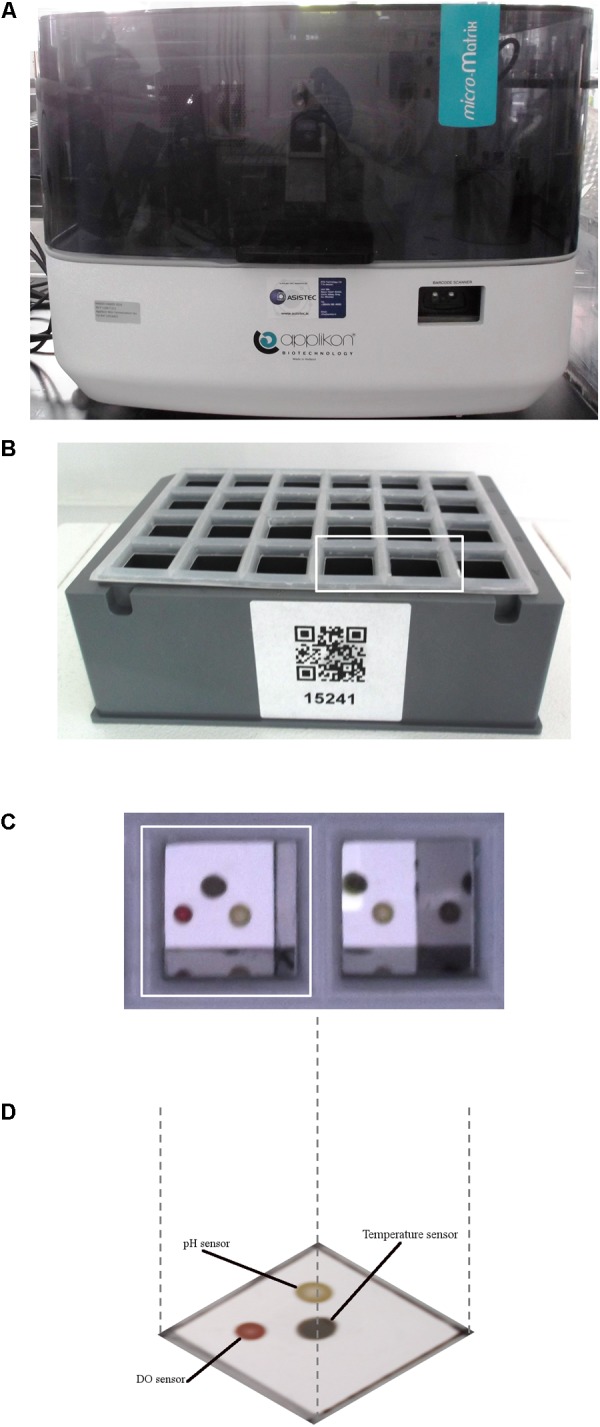
The micro-Matrix fermentation system. **(A)** The micro-Matrix; **(B)** a micro-Matrix 24-well cassette; **(C)** an overview of two wells from the 24-well cassette; **(D)** a representation of a single well of the cassette including the DO and pH sensors and the temperature sensor.

The micro-Matrix (Applikon Biotechnology, Heertjeslaan 2, 2629 JG Delft, Netherlands) was used to model the human distal colon, it is a mini-fermentation system (1–10 mL). The fermentations were conducted in four sealed micro-Matrix cassettes (24 wells/cassette), occupying three wells in each cassette (*n* = 12 samples in total) over 4 days. The medium and carbohydrate were combined in an anaerobic hood an hour before the cassette was inoculated. The medium (5.52 mL) and 480 μl of the thawed standardized inoculum was added to each well in the cassette (initial volume 6 mL). The Time 0 h (T0) sample of 1 mL is taken when setting the cassette up in the anaerobic chamber to leave a total running volume of 5 mL (50% headspace in each well of the cassette). The cassette was then clamped using the transport clamps and transferred to the micro-Matrix. **Figures 1A–D** illustrates the micro-Matrix fermentation system and the 24-well single-use cassette design.

### micro-Matrix Parameters and Sampling

The installation of the filled cassette and the attachment of the relevant parts of the fermenter were performed in compliance with the micro-Matrix manual. Nitrogen gas was under direct control in the machine at 40% output, CO_2_ gas was used for downward pH control, the orbiter was set to 250 rpm and 4 M NaOH was used as a liquid feed as upward pH control. The control set-points for the parameters were pH 6.8 (low alarm 6.6, high alarm 7.1), temperature 37°C (low alarm 35°C, high alarm 38°C), DO 0% (low alarm 0%, high alarm 20%). A fill limit was also placed on the liquid addition of 1.5 mL. One milliliter samples were taken for DNA extraction at Time 24 h (T24).

### DNA Extraction

DNA was extracted from the T0 and T24 samples from each well using the Zymo Research ZR fecal DNA kit (Cambridge Biosciences, Cambridge, United Kingdom). The fecal slurries (1 mL from T0 and T24) were centrifuged (3,000 × *g*, 20 min) to concentrate the cells prior to the addition of the lysis buffer and bead beating. Extractions were carried out according to the manufacturer’s specifications.

### MiSeq Compositional Sequencing

All samples were prepared for MiSeq compositional sequencing using the specifications outlined by Illumina Inc. (Illumina Inc., Cambridge, United Kingdom). The V3–V4 region of the 16S rRNA gene was amplified and Illumina index primers attached in two separate PCR reactions (Klindworth et al., 2013). All PCR reactions and clean up procedures using AMPure XP (Labplan, Kildare, Ireland) were performed as outlined by Illumina Inc. Quantified samples were then sequenced using the Illumina MiSeq (Teagasc Sequencing Center, Moorepark, Fermoy, Ireland).

#### Bioinformatic Sequencing Processing and Analysis

Raw data sequencing reads were quality trimmed using the QIIME suite of tools, version 1.8.0 (Kuczynski et al., 2012). Raw sequencing reads failing to pass the criteria were discarded. Denoising, chimera detection and operational taxonomic unit (OTU) grouping at 97% similarity were performed in QIIME using USEARCH v7 (Edgar et al., 2011). Taxonomic ranks were assigned by alignment of OTUs using PyNAST (Caporaso et al., 2010) to the SILVA SSURef database release 111 (Pruesse et al., 2007). Beta diversity principle coordinate of analysis (PCoA) plots was created using weighted (species abundance) Unifrac metrics with the R phyloseq package (McMurdie and Holmes, 2013). Two samples (one at T0 and one at T24) had to be removed from the study as the number of reads was too low. The number of samples used for sequencing analysis was *n* = 11. Relative abundances ≥0.1% in more than six samples were reported here.

#### Statistical Analysis

The One-way ANOVA test, using SPSS (PASW Statistics version 18), was used to compare the four cassette machine alpha diversities (Shannon and Simpson index) with each other at two time-points. Statistical significance was accepted at *p* < 0.05. One-way ANOVA (SPSS) was also used to compare the four cassettes at the two different time-points at the phylum and genus level. Statistical significance was accepted at *p* < 0.05. Permanova (R-vegan function adonis) was used to analyse the variance from the beta diversity plot.

## Results

We aimed to show that a mini-fermentation system could be used as a colon model. To illustrate this, we tested the same carbohydrate in four cassettes in the micro-Matrix. The results were assessed via the logging information of the system itself and MiSeq 16S rRNA compositional sequencing (culture independent technique). We generated a 16S rRNA dataset consisting of 3,959,193 raw sequencing reads and 3,532,532 high quality filtered reads. There was an average 160,569 sequences per sample (range: 41,563–361,937). Sequences identified from each cassette clustered into 614 OTUs.

### Machine Parameter Logging

**FIGURE 2:**
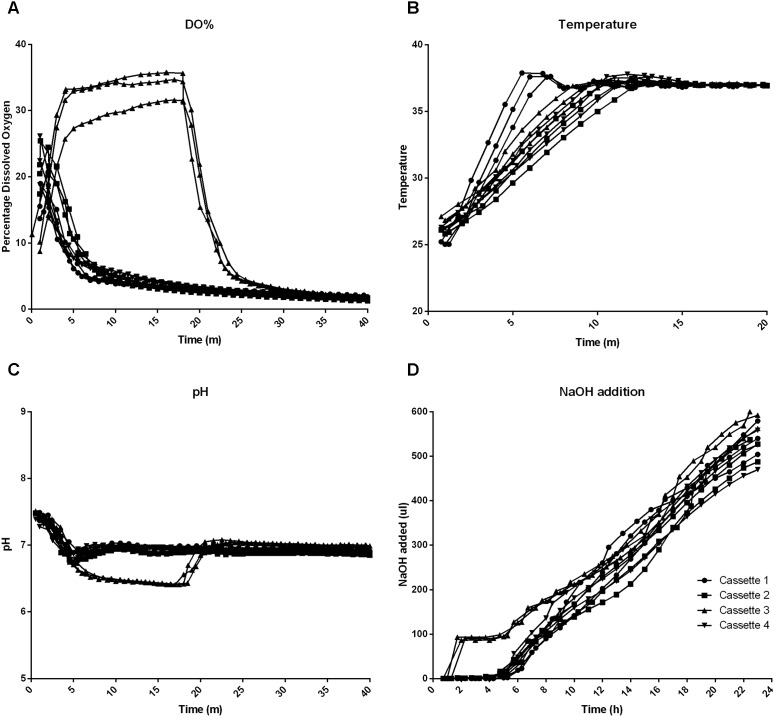
Machine parameters logged over the first 20–40 min of the fermentation **(A–C)** and over the full 24 h fermentation period **(D)**. **(A)** Percentage dissolved oxygen (DO%) **(B)** Temperature **(C)** pH **(D)** Liquid NaOH addition.

The fermentation system monitors a range of parameters during the fermentation run. **Figures 2A–C** illustrates the data logged for %DO, temperature and pH over the first 20–40 m of each fermentation. From the graphs it can be seen that after 10 min all of the wells are virtually identical in their behavior. **Figure 2A** illustrates the monitoring of the DO in the wells of the cassette. Cassette 3 had no nitrogen gas supply for approximately 10 min and the graph clearly shows this but also shows how quickly the machine was able to reduce the DO once the nitrogen supply resumed. The delayed nitrogen supply also had knock-on effects for both the pH and liquid addition of Cassette 3 but this was rectified within 10 min once the nitrogen supply resumed. The DO sensors measured ∼35% air saturation at their maximum in Cassette 3 (during the nitrogen cylinder change) this figure corresponds to approximately a real O_2_ concentration of 7%. After 20 min the air saturation concentration of 2% corresponded to 0.4 ± 1% O_2_. **Figure 2D** illustrates the sodium hydroxide liquid addition to each well over the 24 h fermentation; the wells in each cassette require almost the same amount of sodium hydroxide to maintain physiological pH.

### Alpha Diversity

**FIGURE 3:**
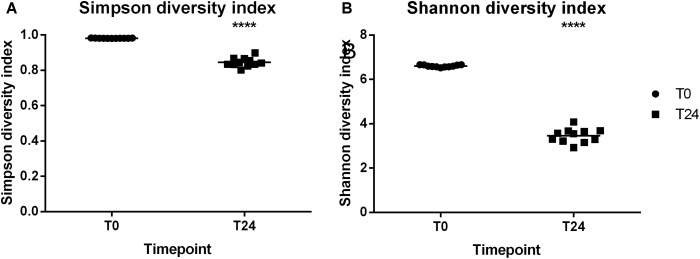
Alpha diversity indices **(A)** Simpson diversity index **(B)** Shannon diversity index. ^∗∗∗∗^*P* ≤ 0.0001.

Alpha diversity describes the diversity within a sample. **Figures 3A–B** illustrates the Shannon and Simpson alpha diversity metrics, respectively, that can be used to assess diversity within a sample. The reproducibility of the fermentation system can be seen with all of the T0 and T24 samples independent of the cassette clustering around the median line. The significant difference between the alpha diversity metrics was between the fermentation at T0 and T24 and not between the cassettes. The major determining factor in the variation between the samples is therefore the FOS fermentation/time-point and not the cassette/fermentation day.

### Beta Diversity

**FIGURE 4:**
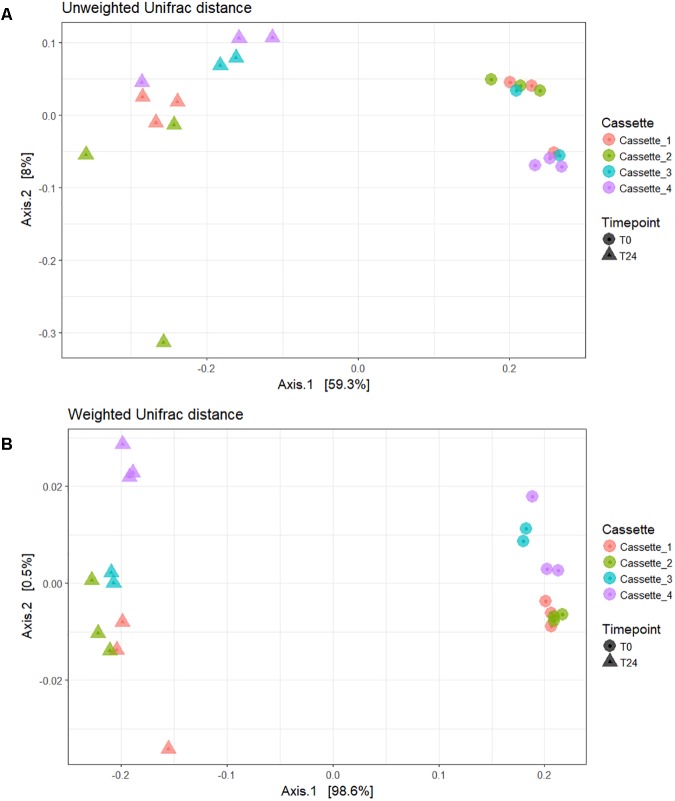
Beta diversity plots. **(A)** Unweighted Unifrac distance; **(B)** Weighted Unifrac distance.

In **Figure 4** the beta diversity (between sample diversity) can be seen. The unweighted Unifrac distance beta diversity plot (**Figure 4A**) showed that the samples are clustered by the fermentation time-point accounting for 59.3% of the variation between the samples. In the weighted Unifrac distance plot (**Figure 4B**) samples clustered by time-point with 99% of the variation between the samples accounted for by the treatment and not the cassette/day. The output of our Adonis analysis using the weighted Unifrac distance matrix was significant (*p* < 0.001) with an R^2^ of 0.98. The large R^2^ accounting for 98% of the variance would indicate that the Time-point (i.e., fermentation of the prebiotic carbon source FOS) is the major contributing factor to the variance seen and not variation between cassettes/fermentations days. The test for the homogeneity of dispersion was also significant (*p* < 0.05) which means we reject the null hypothesis that the sample time-points have the same dispersions.

### Phylum and Genus Level Taxonomic Assignments

**FIGURE 5:**
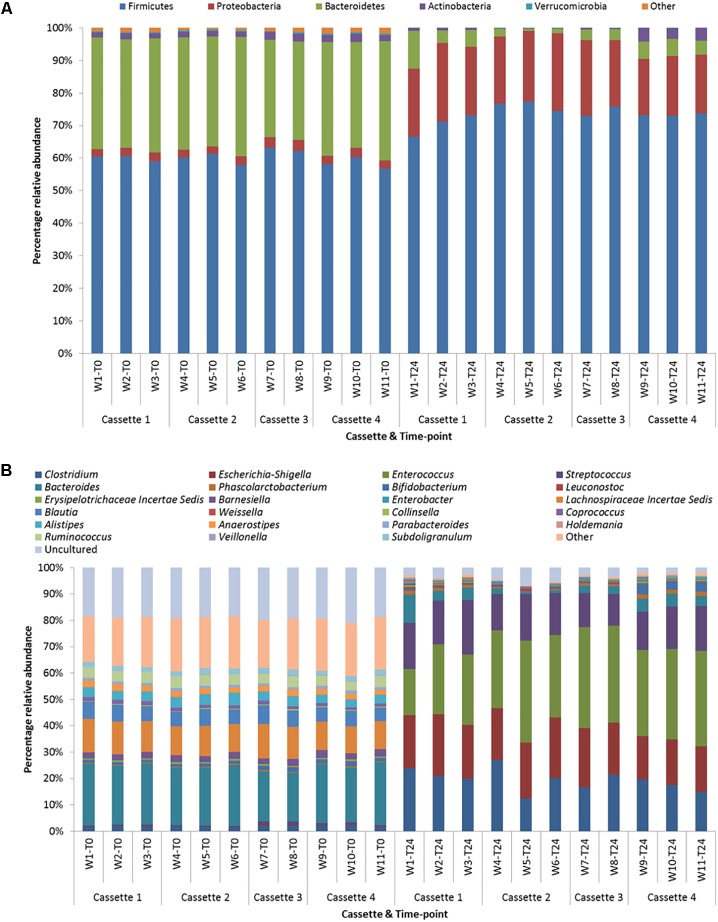
**(A)** Phylum level taxonomic diversity between cassettes and time-points. **(B)** Genus level taxonomic diversity between cassettes and time-points (w = well).

The samples at T0 were dominated by Firmicutes (median 60.3%) and Bacteroidetes (median 34.3%) and at T24 by Firmicutes (median 73.2%) and Proteobacteria (median 20.9%). At the genus level the T0 samples were dominated by *Bacteroides* and *Lachnospiraceae Incertae Sedis* and at T24 by *Clostridium* and *Escherichia-Shigella* and *Enterococcus*. **Figures 5A,B** illustrates the similarity between the time-points irrespective of cassette at the phylum and genus level, respectively. This demonstrates the reproducibility across days/fermentations of the mini-fermentation system. A limited number of statistically significant differences between the cassettes at the two time-points were identified and are listed in **Table 1**. A comparison of the predominant taxa identified at T24 was compared to data we previously generated using an established bench-scale bioreactor batch colon model and are listed in **Supplementary Table 1**. The predominant taxa present in both fermentation systems include the saccharolytic genera *Clostridium*, *Escherichia-Shigella*, *Enterococcus*, and *Streptococcus*. The similarities and overlap in the dominant genera from the fecal microbiota following 24 h fermentations of FOS indicate that the micro-Matrix is accurately replicating results generated in a more standard bench-top fermentation system.

**Table 1:** Statistical comparisons of each cassette.

Taxa	Cassette				P value			
	Cassette 1 (%)	Cassette 2 (%)	Cassette 3 (%)	Cassette 4 (%)	C1	C2	C3	C4
**Phylum level – T0**								
Proteobacteria	2.51	2.42	**3.36**	2.67	^∗∗^	^∗∗^		^∗^
Bacteroidetes	34.25	**35.02**	30.03	34.71			^∗^	
Actinobacteria	1.67	**1.66**	2.39	2.19			^∗^	
RF3	0.69	**0.26**	0.71	1.09				^∗∗^
**Phylum level – T24**								
Proteobacteria	22.03	**22.07**	21.80	17.86				^∗^
Actinobacteria	0.81	0.23	0.49	**3.84**	^∗∗∗^	^∗∗∗∗^	^∗∗∗∗^	
**Genus level – T0**								
*Escherichia-Shigella*	0.10	0.08	**0.41**	0.11	^∗∗∗∗^	^∗∗∗∗^		^∗∗∗∗^
*Bacteroides*	22.81	22.23	**18.56**	22.21	^∗^	^∗^		^∗^
								^∗^
*Phascolarctobacterium*	**0.36^a^**	**0.32^b^**	0.46	0.49			^∗^	^∗∗^
*Lachnospiraceae*						^∗^		^∗^
*Incertae Sedis*	12.25	11.03	**12.67^c^**	**10.55^d^**	^∗^		^∗^	
*Coprococcus*	**1.61**	1.31	1.27	1.17		^∗^	^∗^	^∗∗^
*Alistipes*	3.58	**4.26**	3.68	3.19				^∗^
*Parabacteroides*	1.05	1.04	**0.95**	1.19				^∗^
*Veillonella*	0.11	0.12	0.11	**0.15**	^∗^		^∗^	
*Parasutterella*	0.21	0.20	**0.26**	0.24		^∗^		
*Sutterella*	1.77	1.70	**2.23**	1.80	^∗^	^∗^		
					^∗∗∗∗^		^∗∗∗^	^∗^
*Faecalibacterium*	5.15	**6.68^e^**	5.03	**5.92^f^**	^∗^	^∗^	^∗^	
*Anaerotruncus*	0.20	**0.26**	0.20	0.19	^∗^		^∗^	^∗∗^
					^∗∗^			
*Lachnospira*	1.42	1.25	**1.04^g^**	**0.98^h^**	^∗∗^	^∗^		
*Odoribacter*	0.60	**0.37**	0.63	0.63	^∗∗^		^∗∗∗^	^∗∗∗^
					^∗^		^∗^	
*Prevotella*	1.08	**1.38^i^**	**0.99^j^**	1.32		^∗^		^∗^
RC9_gut_group	**0.75**	0.88	0.86	0.99				^∗^
*Thalassospira*	**0.15**	0.20	0.21	0.25				^∗^
**Genus level – T24**								
*Escherichia-Shigella*	**21.38**	21.29	21.13	17.02				^∗^
*Enterococcus*	**23.55**	33.17	37.60	34.38			^∗^	
*Streptococcus*	**18.28**	15.82	12.39	15.91			^∗^	
*Phascolarctobacterium*	1.07	**0.43**	0.69	1.60				^∗^
						^∗^		^∗∗∗∗^
*Bifidobacterium*	**0.58^k^**	0.15	0.38	**3.47^f^**	^∗∗∗∗^	^∗∗∗∗^	^∗∗∗∗^	

*^∗∗∗∗^P < 0.0001, ^∗∗∗^P < 0.001, ^∗∗^P < 0.01, ^∗^P < 0.05. Note: Values highlighted in bold are responsible for the significant variance in the cassette listed. ^a^Cassette 1 vs Cassette 4. ^b^Cassette 2 vs Cassette 3 and Cassette 4. ^c^Cassette 3 vs Cassette 2 and Cassette4. ^d^Cassette 4 vs Cassette 1 and Cassette 3. ^e^Cassette 2 vs Cassette 1, Cassette 3 and Cassette 4. ^f^Cassette 4 vs Cassette 1, Cassette 2 and Cassette 3. ^g^Cassette 3 vs Cassette 1. ^h^Cassette 4 vs Cassette 1 and Cassette 2. ^i^Cassette 2 vs Cassette 1 and Cassette 3. ^j^Cassette 3 vs Cassette 2 and Cassette 4. ^k^Cassette 1 vs Cassette 2 and Cassette 4.*

## Discussion

The aim of this study was to illustrate the application of micro-bioreactors as batch models of the human colon. The reproducibility of the micro-bioreactor was assessed using a standardized microbiota across four cassettes and using the machine’s own logging information and Illumina Miseq 16S rRNA compositional sequencing. While our “*n*” number for each cassette was small (*N* = 3, total number of samples = 11 per time-point) the reproducibility of the system is still apparent.

This pilot study using the micro-Matrix mini-fermentation system revealed the efficacy of the system in differentiating the initial microbiota from one that has undergone fermentation of a prebiotic carbohydrate. Future studies will need to address the metabolites produced by fermentations in this system, for example short chain fatty acids, and whether or not the results are stable and reproducible across cassettes and runs as demonstrated here with the microbiota. Similar mini-fermentation systems are being developed but involve multiple components that need to be controlled individually (Wiese et al., 2018) rather than a single unified system.

Some of the genera that showed an increase in relative abundance between T0 and T24 include *Escherichia*-*Shigella*, *Enterococcus, Clostridium*, and *Streptococcus*. These facultative anaerobic and strict anaerobic genera contain species that are saccharolytic and have previously shown an ability to utilize FOS as a nutrient source (Hartemink et al., 1995; Roberfroid, 2001; Rossi et al., 2005; Alteri and Mobley, 2012). The increase in the relative abundance of strict anaerobic genera, for example *Clostridium*, during the FOS fermentation is an indication of the micro-Matrix’s ability to maintain an anaerobic atmosphere throughout the 24 h run. Some members of the *Lachnospiraceae* and *Ruminococcaceae* have been shown to have the ability to utilize prebiotic carbohydrates but this capacity is dependent on the degree of polymerisation of the carbohydrate (Scott et al., 2014). This and competition by other saccharolytic genera may account for the reduced abundance of genera like *Coprococcus*, *Roseburia*, *Faecalibacterium*, and *Bacteroides*.

At the start of the fermentation within cassette 3 the nitrogen supply had to be changed and so there was a 10 min period where the machine had no nitrogen supply. The machine’s monitoring and logging system accurately showed the effect that the lack of nitrogen had on the DO percentages and also the pH of the system. The lack of nitrogen so early in the fermentation run, however, had no effect on the fecal microbiota as each cassette was prepared and sealed under anaerobic conditions. The true oxygen percentage never rose above 7% even at its highest during the run and therefore was unlikely to have affected the fecal microbiota.

Preparing samples (DNA extraction, PCR amplification, clean-ups, and quantifications) for 16S compositional sequencing can lead to bias in the 16S profiles (Kennedy et al., 2014). Minute variations in how a sample is prepared/treated can lead to variations within the 16S rRNA profiles. The taxa level variation in the cassettes at the two time-points is limited to a few phyla and genera. The major variation in the samples occurs between Cassettes 1–3 and Cassette 4. This variation between these cassettes may be as a result of using two sequencing runs for the dataset, with all of cassette 4 samples being sequenced in an entirely different reaction. Despite these variations in the relative abundances the reproducibility of the system is evident when looking at the alpha diversity (**Figure 3**) and beta diversity (**Figure 4**) matrices. The samples cluster together in the beta diversity plot with the majority of the variation (99%) in the weighted unifrac PCoA plot coming from the difference between unfermented microbiota (T0) and following fermentation (T24) and not from variations between the cassettes/days the fermentations were carried out.

The small working volume of the micro-Matrix system (1–5 mL) makes it ideal for projects/experiments involving small quantities of test material or studies where fecal samples are difficult to obtain, for example from infants. With each of the 24 wells requiring only 400 μl of slurry it makes it possible for researchers with sample limitations (≤20 mL) to carry out their experiments rapidly but also efficiently. This is a vast improvement on larger systems where working volumes can extend from 100 mL to 1000 mL such that working with small quantities of reagents and inoculum is virtually impossible.

This is the first study to use the micro-Matrix mini-fermentation system as a batch colon model. The use of the FSI in conjunction with the mini-fermentation system ensures a highly reproducible microbiota that can easily be assessed for changes due to treatment. The use of mini-fermentation systems with a relatively large amount of vessels/wells will increase the output of each experiment while simultaneously decreasing the time each experiment will take to complete. This time-saving includes the length of time it takes to sterilize the fecal matter, wash the bioreactors and re-sterilize for the next fermentation which can all take up to 5 h each time which is all negated with the use of the single-use 24 well/bioreactor cassettes. We hypothesize that the reproducibility of the system and high-throughput nature can be extended to investigating changes in the fecal microbiota due to antibiotics, anti-microbials, phage therapies and a whole host of other treatments.

## Author Contributions

MO, MR, and RR designed the experiments. MO was responsible for performing all experiments, analysis and bioinformatics and wrote the manuscript. All authors contributed to interpreting the results, critically revising the manuscript for important intellectual content, and approving the final manuscript.

## Conflict of Interest Statement

The authors declare that the research was conducted in the absence of any commercial or financial relationships that could be construed as a potential conflict of interest.
