# Supplementary material for: The Use of a Mini-Bioreactor Fermentation System as a Reproducible, High-Throughput ex vivo Batch Model of the Distal Colon
Source: Front Microbiol. 2018 Aug 10;9:1844. doi: 10.3389/fmicb.2018.01844 (PMC6096000; doi:10.3389/fmicb.2018.01844)
Supplement: Supplementary file 1 [file Table_1.docx]

| **Supplementary Table 1.** Comparison of the predominant genera identified using the micro-Matrix and the Infors Multifors | | | |
| --- | --- | --- | --- |
| Genus | **T0** | **Infors Multifors FOS T24** | **micro-Matrix FOS T24** |
| *Clostridium* | 1.76% | 12.13% | 19.88% |
| *Escherichia-Shigella* | 0.10% | 35.21% | 20.14% |
| *Enterococcus* | 0.02% | 2.72% | 32.74% |
| *Streptococcus* | 0.66% | 2.44% | 16.20% |
| *Bacteroides* | 22.21% | 0.01% | 3.63% |
| *Phascolarctobacterium* | 0.40% | 0.47% | 0.78% |
| *Bifidobacterium* | 1.23% | 0.52% | 0.52% |
| *Erysipelotrichaceae Incertae Sedis* | 0.60% | 0.02% | 0.34% |
| *Barnesiella* | 2.37% | 0.00% | 0.14% |
| *Lachnospiraceae Incertae Sedis* | 11.47% | 0.08% | 0.21% |
| *Blautia* | 5.73% | 0.01% | 0.18% |
| *Collinsella* | 0.32% | 0.02% | 0.08% |
| *Coprococcus* | 1.31% | 0.00% | 0.01% |
| *Alistipes* | 3.61% | 0.00% | 0.09% |
| *Anaerostipes* | 2.14% | 0.00% | 0.07% |
| *Parabacteroides* | 1.04% | 0.00% | 0.09% |
| *Ruminococcus* | 4.12% | 0.00% | 0.06% |
| *Veillonella* | 0.12% | 0.03% | 0.02% |
| *Subdoligranulum* | 1.97% | 0.02% | 0.06% |

**Note:** Both systems were operated with constant nitrogen sparging, at similar rpm, with sodium hydroxide used to control pH and using the same fermentation media.
